# Life Span Evolution in Eusocial Workers—A Theoretical Approach to Understanding the Effects of Extrinsic Mortality in a Hierarchical System

**DOI:** 10.1371/journal.pone.0061813

**Published:** 2013-04-15

**Authors:** Boris H. Kramer, Ralf Schaible

**Affiliations:** Laboratory of Evolutionary Biodemography, Max Planck Institute for Demographic Research, Rostock, Mecklenburg-West Pomerania, Germany; Institut Pluridisciplinaire Hubert Curien, France

## Abstract

While the extraordinary life span of queens and division of labor in eusocial societies have been well studied, it is less clear which selective forces act on the short life span of workers. The disparity of life span between the queen and the workers is linked to a basic issue in sociobiology: How are the resources in a colony allocated between colony maintenance and reproduction? Resources for somatic maintenance of the colony can either be invested into quality or quantity of workers. Here, we present a theoretical optimization model that uses a hierarchical trade-off within insect colonies and extrinsic mortality to explain how different aging phenotypes could have evolved to keep resources secure in the colony. The model points to the significance of two factors. First, any investment that would generate a longer intrinsic life span for workers is lost if the individual dies from external causes while foraging. As a consequence, risky environments favor the evolution of workers with a shorter life span. Second, shorter-lived workers require less investment than long-lived ones, allowing the colony to allocate these resources to sexual reproduction or colony growth.

## Introduction

Life span is a highly variable trait. Across the tree of life, we find short-lived organisms like flies, which have a life span of less than a week. At the other extreme, long-lived species like elephants, tortoises or humans may live for more than 60 years [1]. Even within taxonomic groups, such as mammals, life span may vary by a factor of 60 [1]. Among insects, differences in the life span of solitary and social species reach a factor of 100 [2]. This significant variation in life span is often explained by differences in the life histories of species. Moreover, life span may be highly variable even within a single species. Among eusocial insects, which are defined by a reproductive division of labor [3], [4], there is a distinct gap between the life span of reproductive queens and the mostly sterile workers [5].

The observed disparity in the life span of queens and workers is determined by differential gene expression within the same genotype [6], [7]. From a diploid egg, worker or queen phenotypes can be reared with divergent demographic properties, such as life span and reproduction. The gap between worker and queen life span is most pronounced in species with caste dimorphism, among which the morphology of the queen and the worker castes differs. Honey bees and ants display a unique pattern of divergence in the life span of different phenotypes. Even among species with less pronounced morphological differences between reproductive and non-reproductive individuals, such as annual social wasps, life span differs depending on the task performed [8].

The maximum life span of eusocial queens is 30 years [2], [9], while the average life span is considerably shorter due to high levels of mortality during colony foundation, especially in species with independent colony founding [10]. Workers may have a life span of less than a month [9], [11]. An extreme degree of variation in life span within the same species is displayed by the invasive fire ant *Solenopsis invicta*. The queen outlives small workers by a factor of 30, even under protected conditions in the laboratory [9], [12].

In addition to this pronounced disparity in queen and worker life span, workers across different species also show a high degree of variation. The mean life span for eusocial hymenopteran workers ranges from 0.1 years for wasps to 1.6 years for ants [2], [13]. Within the Fomicidea, the mean worker life span ranges from weeks to several years [5], [9], [14]. Even within a species, the life span of morphologically similar workers may vary. In the honey bee (*Apis melifera*), diutinus workers (winter workers) may outlive foraging and nursing workers by a factor of four, probably due to adaptations to temperate regions [15], or to task-dependent life history regulation [16]. In species with polymorphic worker castes, life span differs between different castes, either depending on size [12], or independent of size, but determined by the levels of extrinsic mortality associated with the tasks carried out [17]. Compared with solitary insects, the life span of hymenopteran workers is prolonged. For example, the mean life span for solitary insects is 0.1±0.2 years [2], while ant, bee and wasp workers reach a mean of 0.9±1.1 years [13]. The life span of wasp and bee workers differ slightly from those of solitary insects but ant workers have much longer life spans. In the case of ants, the protected subterranean niche might also affect the evolution of life span.

Considerable work has been done to improve our understanding of the evolution of the extended life span and the high fertility of queens in eusocial species, especially in contrast to solitary insects [2], [5], [18], [19], [20]. While researchers have focused on queen or colony life span, the evolution of different phenotypes, including the evolution of worker life span, has received less attention. The disparities in the life spans of queens and workers, or of different worker castes, have been explained by evolutionary theories of aging, and differences in extrinsic mortality depending on the task carried out [11], [16], [17], [21]. Recently, research on aging patterns in social groups has taken the effects of intergenerational transfers and relatedness on life span evolution into account [22](for a review see [23]). In this approach, the strength of selection on mortality, which is usually determined by the remaining fecundity, is additionally modified by transfer effects [22]. This approach can also be applied to social insects [16], [24], and it could be used to explain the task-specific regulation of internal resources found in honey bees [16], [21]. It has been argued that the task-specific regulation of maintenance in honey bee workers could be important for preserving resources at the colony level [21].

Here we want to test the effects of extrinsic mortality on optimal resource allocation to workers in order to explain the evolution of different aging phenotypes in a hierarchical trade-off setting that implements both the individual and the colony levels in eusocial species. First, however, we will describe the important factors for the divergence in life span between queen and workers, and we will show how the evolutionary theories of aging, which connect the levels of extrinsic mortality and life span, may help to explain the evolution of worker life span.

### A) Factors Driving the Differences in Longevity between Queen and Workers

Two main factors, the division of labor and the level of extrinsic mortality, drive the extreme variation in life span within species of social insects. The two factors are interconnected, but in this chapter we want to point out the implications of each perspective. Later we show that the division of labor is the key to distributing the risk of extrinsic mortality among the different individuals in the colony.

#### 1. Division of labor

All eusocial insects are defined by division of labor. The primary division of labor concerns reproduction [25]. In highly eusocial species, a single queen monopolizes reproduction [26] while workers perform tasks related to colony growth and development [25]. The queen lays all eggs which develop into workers, queens and males. Caste determination of diploid eggs, which develop into sterile workers or reproductive queens, is determined by environmental factors. Each individual develops into one caste, with distinguishable stage- and age-specific differences during its life cycle [6], [7]. In highly eusocial species (e.g., Atta leaf-cutting ants), worker castes are distinguished by behavioral and anatomical traits [9], [25]. In addition, workers of the most highly eusocial species exhibit age polyethism, or a temporal division of labor [4], [27], [28]. Safe tasks are performed earlier in life, while risky tasks are delayed to higher ages [29]. The outcome of this process is that, for workers, reproduction and life span are negligible, while the opposite is true for queens [18]. As evolution tends to produce more complex systems, the integration of individuals into colonies adds a new hierarchical layer during the evolutionary transition [30]. Generally, the division of labor in insect societies is comparable to the germ-soma differentiation in multicellular organisms [31]. The co-occurring specialization of individuals leads to a shift in the unit of selection from the individual level to the colony level. Concomitantly, with a shift in the unit of selection, different phenotypes for reproductive and somatic parts of the colony evolved. Consequently, a different role of selection on senescence should exist for the different castes of a colony.

#### 2. Extrinsic mortality

The level of extrinsic mortality of a colony member is directly correlated with its task and caste. The reduced life span of the workers relative to that of the queen in eusocial species has therefore been linked to the differing levels of extrinsic mortality [17], [18], [20].

Workers perform all the risky duties within and outside of the colony. Tasks like foraging, nest guarding and defence entail a higher extrinsic risk of dying than the functions of a queen which resides in the center of the colony. From a colony perspective, the extrinsic risk is distributed to different individuals within the colony via the division of labor. Moreover, social insect workers can distribute the extrinsic risk to different ages in the life cycle by age polyethism [29], [32]: this temporal division of labor improves the survival of the individual by shifting from the performance of safe tasks inside the colony early in life, to the performance of risky tasks like foraging later in life [4], [27], [28].

### B) Evolutionary Theories of Aging for Social Insects

To fully understand why workers die at younger ages than the queen, we need to address evolutionary theories of aging. According to these theories, aging evolves as a consequence of an age-related decrease in the force of selection [33], or by gaining early life benefits at the cost of late life disadvantages [34], [35]. One derived prediction is that castes exposed to increased extrinsic mortality should show an increased rate of aging [2], [35]. Empirical evidence supports this hypothesis [36], [37], [38], [39]. However, recent theoretical studies on the link between extrinsic mortality and life span evolution suggest that more complex mechanisms may be involved than was previously thought [40]. For example, if density dependence acts mainly at older ages, or if survival is density-independent, the effects of extrinsic mortality on selection are reversed or even vanish [40]. It has been argued that differing levels of extrinsic mortality explain the distinct aging patterns of workers and queens in eusocial species [17], [18].

Workers in many species, including the species in focus here, do not reproduce. Thus, it is hard to understand how deleterious mutations in the worker genome, being equal to the queen genome, can accumulate and be propagated in future generations. It has been stated that sterile workers are beyond the explanatory scope of these theories [15], [24], [41].

Kirkwood’s disposable soma theory [34] explains how trade-offs between maintenance and reproduction lead to certain life histories, but it has the same difficulties in explaining the life histories of individuals within colonies, the unit of selection. When we look at the individual level, there appears to be no trade-off [42], [43]: reproductive capacity and life span are low for workers, while the opposite is true for queens, which makes eusocial species a striking exception to the rule [18]. It has also been argued that the fecundity/life span trade-off is reversed [20], or that the variation within species goes against the normally observed life history trade-off between reproduction and longevity [18]. Individuals are embedded in colonies, and the exclusive consideration of those individuals can be misleading.

From a “superorganism” perspective [44], [45], the unit of selection should be transferred to the colony level. Aging theories would then be able to explain the specific life histories of a colony, including life span, but do not account for the individuals within the colony: at the colony level, an investment in maintenance can be seen as an investment into workers that provide most of the functions needed for colony maintenance. Thus, it has been argued that workers are disposable at the colony level [46], and that the disposable soma theory can be meaningfully applied to social insects to explain selection for resource investments in workers [41]. According to the disposable soma theory [34], long-lived colonies should invest more into somatic maintenance than short-lived ones. But a further trade-off appears: resources for somatic maintenance at the colony level can be invested in either quality or quantity, a trade-off that is comparable to the trade-off between quality and quantity of offspring [47], [48]. Here we define quality as an investment in vitality, which defines the capacity of an organism to withstand destruction. It can be measured at any age by the chance of surviving to the next age [49], [50]. An investment in quality is continuous, and can be independent of the size of an organism. High quality generates high investments in the physiological maintenance of workers, which would result in long-lived workers. Investing in quantity would help to build the workforce by adding more workers with lower quality. These two possible ways of investing in somatic maintenance at the colony level may be equally favorable. However, the trade-off between quality and quantity of maintenance is not implemented in the disposable soma theory. In order to understand how selection for different life spans in social insects may operate, it is necessary to reformulate our understanding of the mechanisms that shape trade-offs in hierarchical systems.

Different levels of extrinsic mortality lead to the differences in the life span of the queens and workers of a single species. But an evolutionary mechanism by which extrinsic mortality leads to different aging phenotypes in a colony setting has not been proposed.

The goal of this article is to propose a mechanism by which an optimal adjustment of worker life span/quality in response to extrinsic mortality can be selected for at the colony level. For this purpose, we will develop a hierarchical trade-off model incorporating the individual and the colony level. This model will then be tested by extending a model used for annual eusocial species [51] with the hierarchical trade-off. While focusing on highly eusocial species with no conflict over reproduction, we will test the hypothesis that worker life span reduction may be adaptive at the colony level, and serves to reduce the loss of resources that could otherwise be invested in sexual reproduction. This would mean that the reduction of the life span of the worker relative to that of the queen may not be an outcome of weaker selection at older ages, as has been proposed in the evolutionary theories of aging, but may instead be a result of a quantity/quality trade-off that is actively selected for.

### The Hierarchical Trade-Off in Eusocial Species: A Novel Approach

Focusing on the trade-off between reproduction and maintenance [52], both hierarchical levels (individual and colony level) may have different implications.

**Figure 1:**
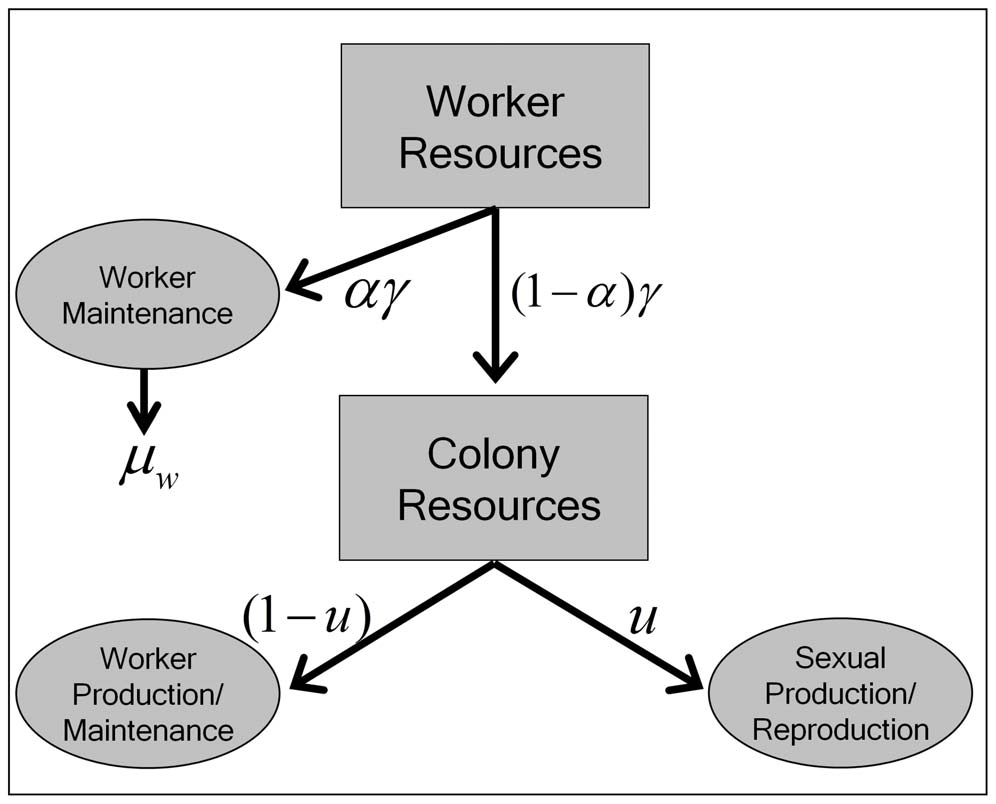
Hierarchical trade-off model for eusocial species. Simplified hierarchical trade-off with a focus on workers for eusocial species, including two trade-offs at the colony and the individual levels. Arrows indicate resource flows. Resources are obtained by workers that do not reproduce and are allocated toward worker maintenance (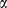) and/or the colony (1−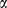). At the colony level, resources that are not consumed by workers can be allocated to sexual reproduction (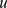) and/or maintenance (1−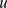), such as the production of new workers or different levels of worker quality.

1. At the individual level, investment in maintenance sustains bodily functions. This prevents organismal deterioration, and thus increases life span and future reproductive success. The level of investment in worker maintenance is low, which leads to a short life span. Workers in many insect societies do not reproduce, or have a reduced ability to do so. Thus, there is no trade-off between maintenance and reproduction for workers. The queen distributes high levels of resources to maintenance and reproduction, which leads to a long life span and high fertility. Reproduction by the queen leads to the production of new individuals (workers, queens or males).
2. At the colony level (Fig. 1), sexual reproduction is accomplished by rearing queens and males. Male eggs may be produced by workers in several species [53]. Virgin queens are produced from eggs laid by the queen under specific environmental conditions, which trigger gene expression that differs from the gene expression of workers [6]. Maintenance at the colony level includes investment in workers and results in building a workforce (colony growth) during the ergonomic phase of the colony. Alternatively replacing dead workers or changing the quality of the workers also represents maintenance on the colony level (see Fig. 1). These investments increase colony survival by enhancing its protection and increasing resource availability and generally help to buffer the environment [54], [55], [56]. It should be noted, however, that having high levels of maintenance at the colony level does not necessarily mean that the investment per worker increases. Thus, having high levels of worker maintenance does not always produce the same outcome as having high levels of colony maintenance. Empirical data suggest that species with bigger colonies (higher investment in colony maintenance) have shorter-lived workers (low levels of investment in worker maintenance) [13].

The resource flow of a colony (Fig. 1) is controlled by the influx and allocation within the hierarchical organization of the colony: resources are collected by foraging workers and brought into the colony. Resources that are not consumed by foraging workers are transferred to the colony to supply non-foraging workers, the brood and the queen. The economical use of resources for worker maintenance and the foregoing of worker reproduction increase the amount of resources available at the colony level. These resources can be channeled to either the maintenance or the reproduction of the colony. In short: if fewer resources are needed for maintaining individual workers, more resources are available for the reproduction-maintenance trade-off at the colony level (Fig. 1).

With this conceptual framework, it is possible to elaborate the benefits for the colony achieved by a reduction in investments in individual workers, or a shift from quality to quantity. Colony-level selection that acts to maximize colony fitness should shape investments in workers depending on the age independent mortality risk. These investments could be channeled in individual maintenance, thus achieving high levels of repair. Alternatively, changes in the initial investment, reflected in the body size of workers, could be modified. This can happen independently of worker quality. Each worker that dies of extrinsic causes means a loss of the resources already invested in the worker, as well as a loss of potential future work. An optimized adaptive demography could entail a lower investment in workers, which would reduce the individual life span, but would simultaneously reduce the potential loss of investments due to the high extrinsic mortality risks of the workers. The foraging worker needs to amortize the costs put into it from the colony.

### Previous Model Specification

To show the effects of extrinsic mortality on the hierarchical trade-off in colonies, we modified an optimization model by Macevicz & Oster [51] used for annual eusocial species. The original model offers a simple solution for incorporating individuals into a colony. The model runs for one season (200 days), producing a fitness value. This approach incorporates the effects of different allocation strategies into worker maintenance, but it avoids having to take into account the complicated allocation strategies over several seasons found in perennial species. The model assumes a bang-bang strategy: at the beginning of the season, all resources are invested in the production of workers (ergonomic phase). At the switching time (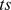), all of the resources are invested in the production of sexuals. The model does not explore reproduction trade-offs between male or female reproductives; the sexuals produced represent queens, and will subsequently be called queens. The model by Oster & Wilson [51], which was also used by Poitrineau et al. [57], consists of two coupled differential equations:
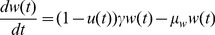
(1)

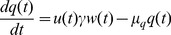
(2)


Time (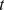) determines the number of workers (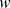) and the number of queens (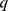). The colony cycle starts in the spring with one inseminated and hibernated female that acts as a worker starting to forage (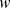
(0) = 1). All of the workers leave the colony to forage and return resources (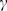) to the colony, which can then be allocated to colony growth and maintenance through the production of new workers or queens. The allocation function at the colony level (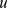) only take values of zero at the beginning of the season, meaning that all of the resources are invested in the production of workers (ergonomic phase); and one after the switching time (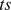), which assumes that resources are exclusively invested in queen production at the end of the season (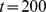), an approach that has been criticized not to capture the biology of annual wasps [57].This parameter represents a trade-off at the colony level, where resources can be allocated to either growth/maintenance or to sexual reproduction. This approach has been criticized for not capturing the biology of annual wasps [58]. Since the approach used here only asks about the effects of maintenance investments under different levels of extrinsic mortality, this simplification is valid, and it also avoids other assumptions about the timing of investments in sexual offspring or the workforce. The mortality of workers (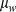) and queens (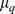) is constant over time. Fitness is measured by the number of queens alive at the end of the season (see Poitrineau et al 2009 for results and discussion of the model).

To incorporate a density-dependent logistic growth for the colony, Poitrineau et al. [57] added the following term (eq. 3), which leads to a reduction in foraging efficiency with increasing colony size, where
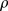
represents density dependency:
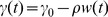
(3)


### Our Extensions

The extensions to the previous model focus on the evolution of worker life span as an adaptive response to the age-independent mortality risk. Fig. 1 shows a schematic representation of the extended model. Colony fitness, measured as the number of queens produced at the end of the season, is the measure to be optimized. To account for the lower (individual) level trade-off, at which point the decision about the amount of resources that are used to maintain the workers is made, an allocation parameter (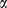) was added to the resource term:
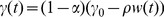
(4)


This leads to a reduction in the resources brought back to the colony by each individual. The amount of resources invested in worker maintenance increases with 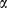, generating a decrease in intrinsic mortality for the workers (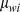). Mortality is composed of an intrinsic mortality term and an extrinsic mortality term for both queens (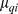;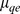) and workers (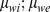). The extrinsic mortality for the queens is important because, once produced they leave the colony and need to survive until the end of the season to be included in the fitness measure. Extrinsic mortality of foraging workers and queens that left the colony is equal (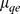 = 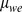).
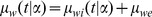
(5)

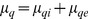
(6)


The intrinsic mortality of the queens (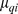) is fixed, and it is also the lowest value that worker mortality (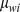) can reach, since queens usually represent the phenotype with the longest life span. Since the costs of worker maintenance need to be linked to the reduction in daily foraged resources, the following equation was included in the model. It assumes that reaching the same low mortality as the queen would consume all of the resources gained while foraging. Also included is the reduction in foraging returns caused by the density dependency (Eq. 3), which leads to lower investments in workers when the foraging returns decline. The queens that have been reared to maturity leave the colony, thus they do not take resources from the colony for maintenance.
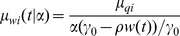
(7)


It should be noted that, in the extreme case in which *α* = 1 (i.e., all foraged resources are kept by the foraging individual) and w(t) = 0 (i.e., there is no reduction in foraging efficiency), the intrinsic mortality for workers is as low as it is for the queen. This would lead to a solitary life for each individual, because no resources would be transmitted to the colony. With reduced investment in workers, the mortality increases, but the resources of the colony also grow.

With the changes mentioned in (3–7), the model equations become:
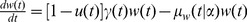
(8)

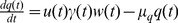
(9)


The number of queens at the end of the season (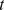 = 200 days) is the fitness value, which is optimized by changing the switching time (*ts*) and worker maintenance (*α*) (Fig. 1). The optimization was done using the optim function with the L-BFGS-B method from the R stats package [59].

## Results

**Figure 2:**
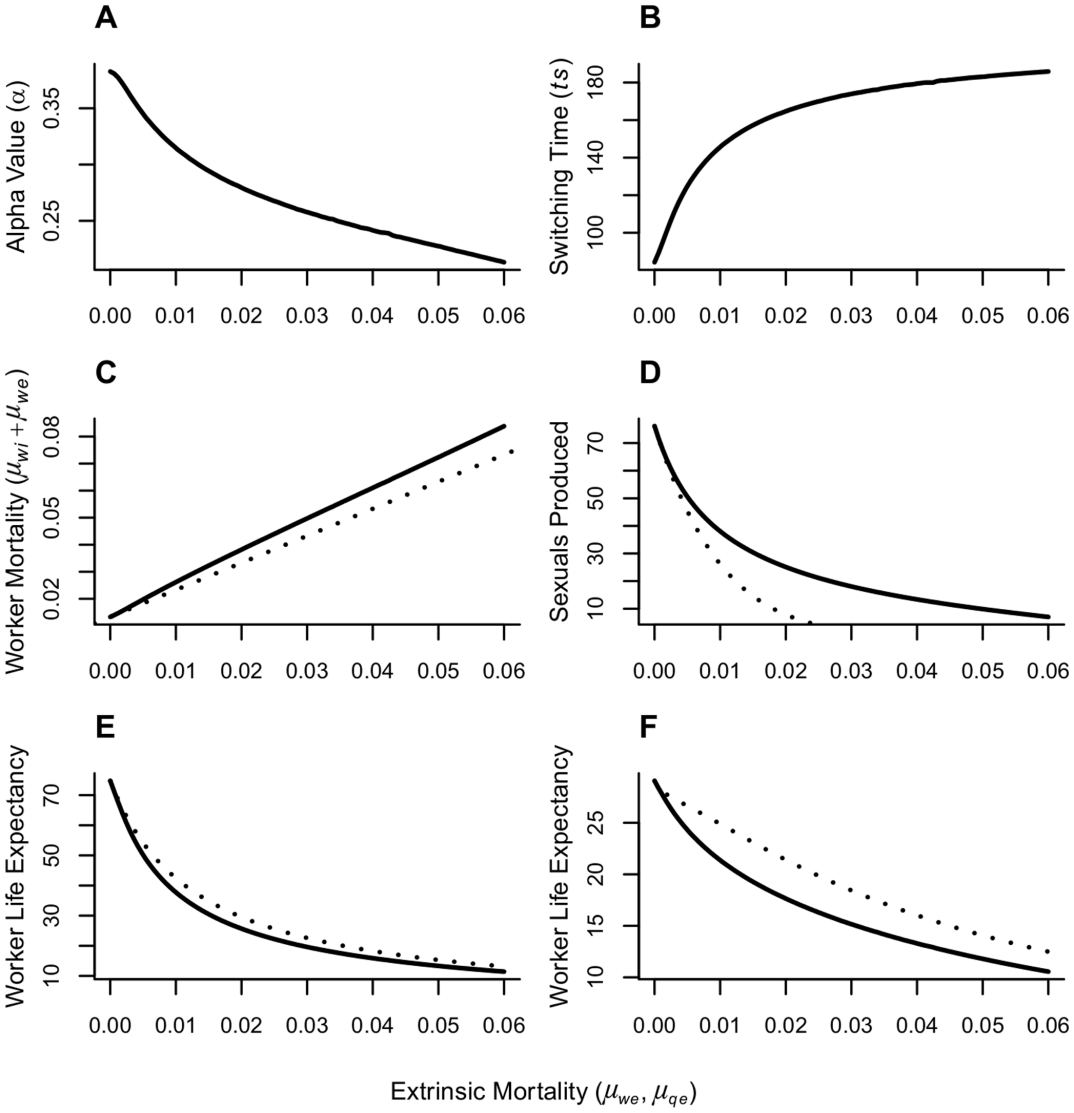
Model results under different levels of extrinsic mortality. The horizontal axis represents the values of extrinsic mortality used to run the model(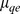;). A) and B) show the optimized parameters (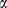, 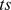,) from our model. In C)–F) the solid lines indicate the results when the optimal values of 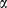 and 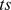, The dashed lines indicate results if the colony did not change the maintenance investments (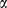) or the switching time (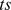) with increasing extrinsic mortality (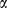 = 0.38 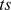 = 84). A) Optimal investment into workers (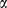) decreases with increasing extrinsic risk. B) Denotes the switching time (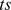), where the colony switches to the production of sexuals. C) The number of sexuals alive at the end of the season (maximized by finding optimal values for switching time (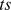) and maintenance investments into workers (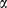)) decreases with increasing extrinsic mortality. D) Worker mortality combines intrinsic and extrinsic mortality (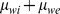). The dashed line denotes the increase of extrinsic mortality. The difference between the dashed and solid lines shows the effect of the changing investment in worker maintenance. E) Worker life expectancy at the beginning of the season with different levels of extrinsic mortality. F) Worker life expectancy at switching time. At switching time, the worker population reaches its maximum. The difference in worker life span between e) and f) is due to the reduction of foraged resources caused by density dependency. Used parameters: 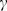 = 0.15, 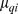 = 0.005, 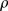 = 0.0024, 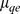 = 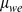 = 0–0.06.

The results of the model show that the negative effects of increasing extrinsic mortality on colony fitness (Fig. 2D) can be attenuated by adjusting the quality of workers (Fig. 2A) via an evolutionary process. This indicates that the change in worker maintenance is adaptive at the colony level because it is able to buffer the reduction in colony fitness deployed by increasing levels of extrinsic mortality. Changes in the life expectancy of workers are driven by density dependency and extrinsic mortality, but are attenuated due to reduced maintenance investment in higher extrinsic mortality settings. Fig. 2 shows the results of the optimization model (parameters: 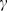 = 0.15, 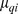 = 0.005, 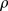 = 0.0024). For 100 levels of extrinsic mortality (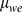 = 0–0.06), the optim function is used to find optimal values for maintenance (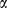) and switching time (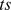) that maximize the number of queens alive at the end of the season.

### Investment Into Workers

Fig. 2A shows that optimal investment in workers (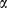) decreases with increasing extrinsic mortality risk. This reduced investment in worker maintenance increases the intrinsic mortality of workers and decreases life expectancy (Fig. 2E/2F). On the other hand, changing maintenance investment reduces the loss of resources for the colony due to the death of workers, because the daily need for resources by the workers decreases. The reduced investment distributes the resources that the colony gains through foraging to more individuals, which in turn die earlier. The dashed line indicates the fixed 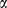-value used to show the divergence in colony fitness (Fig. 2D) and worker mortality (Fig. 2C).

### Worker Mortality

Fig. 2C shows the increase of total worker mortality with increasing extrinsic mortality. Total worker mortality is composed of intrinsic and extrinsic mortality (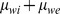). The reduction in the investment into worker maintenance increases worker mortality (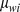), in addition to the rise in extrinsic mortality (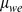). The rise in extrinsic mortality alone is indicated by the dashed line. The reduced investment in workers under higher levels of extrinsic mortality keeps the net energetic efficiency of a worker from becoming negative, but it is still decreasing (not shown).

### Sexual Reproduction (Fig. 2D)

With increasing extrinsic mortality, the number of queens alive at the end of the season decreases. The dashed line indicates the queens produced if there were no change in investment (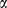) or switching time (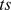) with increasing risk, while the solid line shows the results from the optimization. The difference between the two lines represents the fitness benefits of changing strategies with increasing extrinsic risk. It shows that a reduced or adapted investment into workers and a change in switching time are more efficient than keeping the strategy that is favorable in low-risk environments. Due to the interaction of 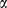 and 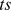, we also run the model with either 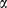 or 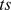 values obtained from the optimization, while the other parameter (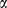 or 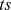) obtained in the low extrinsic mortality setting was held constant. The effect of 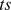
on sexual reproduction is stronger than the effect of 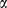, but the effect of 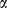
increases with increasing extrinsic mortality explaining up to 44% of the fitness differences. Consequently, if colonies are able to adapt investment into workers to extrinsic risk, they are capable of surviving at higher levels of extrinsic risk.

### Switching Time

The optimal switching time to sexual reproduction is earliest in the low risk environment, and it increases with increased levels of mortality (fig. 2B). In low risk environments, the switching time is driven by density dependent effects: low extrinsic mortality increases the number of workers. This leads to a decrease in the resources brought back by each individual, making it efficient to produce queens early. Additionally, queens have a low intrinsic risk of dying (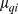 = 0.005), which favors their early production. Queens increase the fitness of the colony only if they survive until the end of the season. In high risk settings, queens produced early cannot survive until the end of the season, and the density-dependent effects do not reduce the foraging efficiency of workers. As a consequence, the colony switches later to the production of queens.

### Worker Life Expectancy

Fig. 2E shows worker life expectancy at the beginning of the season under different levels of extrinsic mortality, while Fig. 2F represents the life expectancy at switching time (*ts*), when the worker population reaches its maximum. The dashed line indicates worker life expectancy without an adjustment to increasing levels of extrinsic mortality. The solid line represents the life expectancy using the optimal alpha value (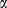). The divergence between the dashed and the solid line represents the changes in life expectancy due to changes in maintenance investments. At the beginning of the season (Fig. 2E), the life expectancy of workers is ∼74 days (calculated by (−1/log(survival))) with low extrinsic mortality, and it reaches ∼11 days under the highest level of extrinsic mortality (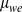 = 0.06). The difference in life span due to changes in investment reaches ∼5 days. At switching time (Fig. 2F), life expectancy is ∼29 days and it declines to ∼11 days at high levels of extrinsic mortality. The difference between Fig. 2E and Fig. 2F is driven by density dependency, which also reduces the amount of resources available for worker maintenance. Reducing the effects of density dependency yields the same results for worker life expectancy, but leads to the production of higher numbers of workers and queens. The changes in life expectancy due to changes in maintenance investments are minor compared to the effects of density dependency and extrinsic mortality, but they nonetheless lead to major changes in colony fitness (Fig. 2D).

For a comparison of the model we used parameters from Poitrineau et al. [57]. Their parameters (productivity 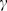 = 0.035, mortality of queens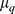 = 0.01, size dependency factor 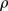 = 0.005) yield the result that no queens are produced until the end of the season (result not shown). The foundress acting as a forager dies without the production of new workers. Since productivity now incorporates maintenance costs, which were not included in the earlier models, productivity needs to be raised. We have also reduced the intrinsic mortality of the queen because we added extrinsic mortality separately, which leads to lower, equal and higher mortality, as used in [57].

High levels of density dependency (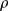
>0.05) have the opposite effect (not shown) on the investment into workers. Low levels of extrinsic risk lead to high worker survival, and the individual foraging returns decline due to density dependence. The optimal strategy is then to invest less into workers and increase their mortality in the low-risk environment to overcome the effects of density dependence. Lower levels of density dependency (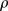
<0.002) lead to unreasonable large colony sizes and high numbers of sexuals produced at the end of the season (>500). The reduction of worker maintenance with increasing extrinsic mortality follows the same trajectory, but is less pronounced.

## Discussion

The presented optimization model shows that a reduction of worker life span may be an adaptive response of the colony under the influence of extrinsic mortality. The fitness of the colony improves by a risk dependent investment into its workers. This indicates that the investment into workers should be under strong selection. The hierarchical trade-off within a “superorganism” can explain how extrinsic risk alters resource flows within colonies. Additionally, the integration of individual-level trade-offs within colony-level trade-offs explains why workers seem to have “reversed” trade-offs. Under increasing levels of extrinsic mortality, this leads to a contradictory process in which a shorter worker life span, as a result of economic resource management, leads to a higher fitness for the colony. This occurs by reducing the risk of losing investments made into individuals and simultaneously offers the option to invest the saved goods into building a stronger workforce or to produce more sexual offspring. The regulation of worker life span may have evolved as an energy-saving mechanism at the colony level [24], [41].

Even though the presented model represents the biology of an annual species following a bang-bang strategy, we assume that the economical considerations underlying the hierarchical trade off presented here apply to all social insects.

The hierarchical trade-off shown here can be used to explain why (via environmental selective pressure) the colony is capable of protecting resources by modifying the life span of workers internally. The model has implications for the investment into worker quality under different levels of extrinsic mortality. The results show that under certain conditions it is useful for the colony to be parsimonious with its resource investments into individual workers. The lower the costs of a worker in an environment with a high age independent mortality risk, the higher the chances that the worker will be capable of amortizing its own production costs. At lower levels of extrinsic mortality higher levels of maintenance investments into workers are favorable. Colonies would not survive without adapting the maintenance investment to the different levels of extrinsic risk because the number of queens produced at the end of the season could drop below one individual.

High risk environments would have a more negative effect on colony fitness if the extrinsic risk faced by individuals, could not be distributed to different castes. This result is consistent with Michod’s [60] finding that the conversion from cell groups to multicellular organisms implies a shift in the level of selection to the colony, as a specialization of reproductive and vegetative functions is needed. Maintenance investments are channeled into the somatic maintenance machinery of the colony, instead of into the individual worker [24], [41]. Resources for somatic maintenance at the colony level could be invested into the quality or the quantity of the workers. Since individuals are loosely integrated and do not gain value throughout their life time (no learning), they are easy to replace, given that production costs are low. Most social insects form spontaneous task groups instead of persistent groups [61], but a task specialization of individuals is possible [62]. This organization ensures a high degree of flexibility, while also allowing for an exchange of individuals without the attenuation of individual efficiency. High levels of individual flexibility are a result of low integration. This effect can be seen in simple multicellular organisms [63] with loosely integrated cells and a high level of regenerative ability. If groups (of cells or workers) were to persist over time and increase in efficiency, an exchange of individuals could lead to a decrease in group efficiency, making it important to maintain the members of the group.

The model developed here shows clearly that the colony should keep the loss of resources low by economizing the investment in individual workers. With the proposed mechanism, extrinsic mortality acts directly on worker life span evolution.

As a proximate mechanism for the regulation of aging in honey bees, the lipoprotein vitellogenin has been proposed [41]. It has been shown that the regulation of aging via vitellogenin also controls the depletion of nutrients in honey bees [41]. Under high levels of extrinsic mortality, a colony with a mutation leading to a reduced investment in workers through a change in the regulation of vitellogenin could outcompete a colony without this mutation, because more resources would be available. This trait would be directly selected for, and could become fixed in a population facing high levels of extrinsic risk. In a mutation accumulation framework, the force of selection declines with age, and deleterious mutations leading to a reduced life span would not be selected against. This is the common explanation for the link between extrinsic mortality and life span. We argue that the mechanism proposed here is a much simpler evolutionary process by which worker life span could be shaped. The reduction of worker life span would be adaptive at the colony level, since it increases the fitness of the colony. Additionally, the model presented combines energetic benefits and the effects of extrinsic mortality to an entity that can be optimized by natural selection.

### Empirical Evidence

The model shows that a reduced investment in workers with increasing extrinsic mortality is economical for the colony. But the model fails to answer the question of which physiological adaptations would lead to a reduction of resource loss due to the extrinsic death of workers. Here we will discuss several adaptations in colonies of social insects that minimize resource loss caused by age-independent mortality. Generally, the model presented needs data on energy costs for workers depending on caste, task and extrinsic hazard, as well as data on longevity and maintenance costs. Currently, however, the data records in the literature are very weak. Nevertheless, there are some examples that might lead to future research.

One possible approach is to compare physiological adaptations of closely related species: *Lasius niger* and *L. flavus* are closely related, but workers experience different levels of extrinsic mortality. *L. niger* is a synanthropic species with a broad food spectrum [64]. This leads to higher extrinsic mortality during foraging. *L. flavus* has a mainly subterranean way of life, including trophobiosis with subterranean aphids [64]. This leads to a lower extrinsic risk for foragers. Following from the model, *L. niger* should invest less in individual workers than *L. flavus.* A comparison of worker life span data reveals that *L. niger* workers live one to two years [65], whereas *L. flavus* workers may live up to 10 years [66]. A comparison of biomass reveals that *L. niger* workers are lighter (0.58 mg dry weight) than workers of *L. flavus* (0.86 mg dry weight) [67]. Following the predictions from our model, the species with the higher mortality risk reduces the initial investment/production costs for workers relative to the species with the lower risk. Thus, losing one worker to an extrinsic risk is not as costly for *L. niger* as for *L. flavus*. Additionally, metabolic costs represent a measure of daily costs for the individual, including maintenance/repair, which determine the quality of the individual. The smaller workers of *L. niger* show lower respiration rates per mg of biomass (1.08 mm^3^ O_2_ mg^−1^ h^−1^) than *L. flavus* (2.04 mm^3^ O_2_ mg^−1^ h^−1^) [67]. For an interspecies comparison, the difference in the metabolic rates of *L. niger* and *L. flavus* can be interpreted as higher maintenance costs for the species with the lower extrinsic mortality as predicted by the model.

The fire ant *Solenopsis invicta* has a polymorphic worker caste. The head width of large workers is twice that of small workers. Large workers live 50% longer than minors in treatments with 24°C. Indeed, their maintenance costs measured as respiration rate per mg tissue at 24°C of 0.9 µl O_2_ h^−1^ mg^−1^ are lower than those of smaller workers 1.55 µl O_2_ h^−1^ mg^−1^ [12], but the absolute economic costs per individual are, due to their greater size, equal to those of at least four small workers [12]. The large workers only forage for the last 25% of their lives, whereas small and medium-sized individuals do so for about 50% at the end of their lives [12]. This again shows that the productivity/cost ratio for the worker is under strong selection. This process keeps colony efficiency at high levels. The life histories of individuals within the colonies are adjusted to keep resources within the colony. In this case, the timing of foraging in the life cycle is more limited to later ages among the larger, more expensive workers than among smaller workers, which reduces the potential loss generated by extrinsic mortality.

In *Solenopsis invicta* and other species, the first workers produced at the start of colony development tend to be smaller (cheaper) and shorter lived (5%) than the individuals produced later in the colony development [68], [69]. In species with single founding queens, this leads to a higher number of workers that can be reared from the limited resources. This adaptive process at the colony level maximizes early colony productivity, while increasing individual efficiency via the parallelization of tasks, and spreading the risk of forager mortality [70]. In addition, these early workers develop faster, which is also important at the early stages of the colony [71]. These findings suggest that there is a quality-quantity trade-off within the maintenance investments at the colony level, and that it is selected for in order to increase the fitness of the colony.

Several studies have also shown that the quality of the workers declines when they perform more dangerous tasks. In species with age polyethism individual nutrient stores are depleted during more risky tasks to keep resources within the protected colony and to reduce the chances of losing those resources due to the death of foragers [41], [72], [73].This process includes a reduction in the fat body, water content, as well as in immune cell count [72]. Thus, the onset of foraging and not age is the best predictor for life span in the honey bee [16].

If the immune response is triggered (without an actual infection) in several individuals of a single bumblebee colony, the production of sexuals is highly reduced, without individuals getting sick [74]. This shows that the somatic maintenance of workers is turned down to a level that keeps the ratio of productivity to costs high. From a colony perspective, it means that resources are kept within the protected colony to avoid loss. But more costly mechanisms, such as the immune system, can still be activated if needed.

The weaver ant *Oecophylla smaragdina* shows a bimodal size distribution, which is correlated with a pronounced division of labor [9], [17]. Minor workers stay within the nest, while major workers attend to more risky tasks. In laboratory experiments with a low level of extrinsic risk, minor workers show a significantly higher survival probability than the major workers, even though the majors have three times the body mass [17]. This shows that the level of extrinsic mortality may be more efficient in shaping survival than body size and metabolism in weaver ants, and that life span may be less affected by physiological constraints [17]. The quality (expressed as life span) of the workers seems to be independent of size. Generally, different morphological worker castes tend to accomplish specialized tasks more efficiently [75]. If the specific task or tasks of a morphological caste are linked to other levels of extrinsic mortality, the investment in quality seems to be selected accordingly. This is in line with the framework of the hierarchical trade-off presented here.

Our simple model is just a first step to understand trade-offs in hierarchical systems. Even if the model represents a special case within the social insects (annual eusocial species and the assumption of a bang-bang strategy instead of a graded transition [57]), we are sure the economical consideration within can be expanded to perennial species, as our empirical evidence section shows. A more general model incorporating a wider range of the variety of lifestyles found within the social insect would be a further step to understand the implications of the presented process but so far the data on maintenance cost and quality quantity trade-offs of social insect worker are not available und thus we decided to start with a simple but several times evaluated model. The general nature of the model reduces the assumptions that need to be made. At the same time this offers the opportunity to extend the specification of the model. The model predicts the optimal investments in workers but does not cover for example life span evolution of queens or the case of multiple worker castes. Moreover, including the production costs for different worker castes/sizes and adding age-dependent mortality could help us to better understand how colonies are able to distribute extrinsic risk among their members. This approach could also be extended to cover worker reproduction and its effects on colony fitness (costs for ovary development, conflict over reproduction). However, in spite of these limitations, this model shows how extrinsic mortality might feed back to the colony, and how selection could work to increase colony fitness under certain conditions.
